# Supplementary material for: Plant dominance in a subalpine montane meadow: biotic vs. abiotic controls of subordinate diversity within and across sites
Source: PeerJ. 2018 Sep 21;6:e5619. doi: 10.7717/peerj.5619 (PMC6152469; doi:10.7717/peerj.5619)
Supplement: Supplemental Information 1 [file peerj-06-5619-s001.pdf]

Supplementary Figure 1. Mean (± standard error) subordinate species' cover, richness, evenness and diversity across plant removal treatments (*Potentilla* removal, *Festuca* removal, no removal control) for growing seasons 2013 and 2015.

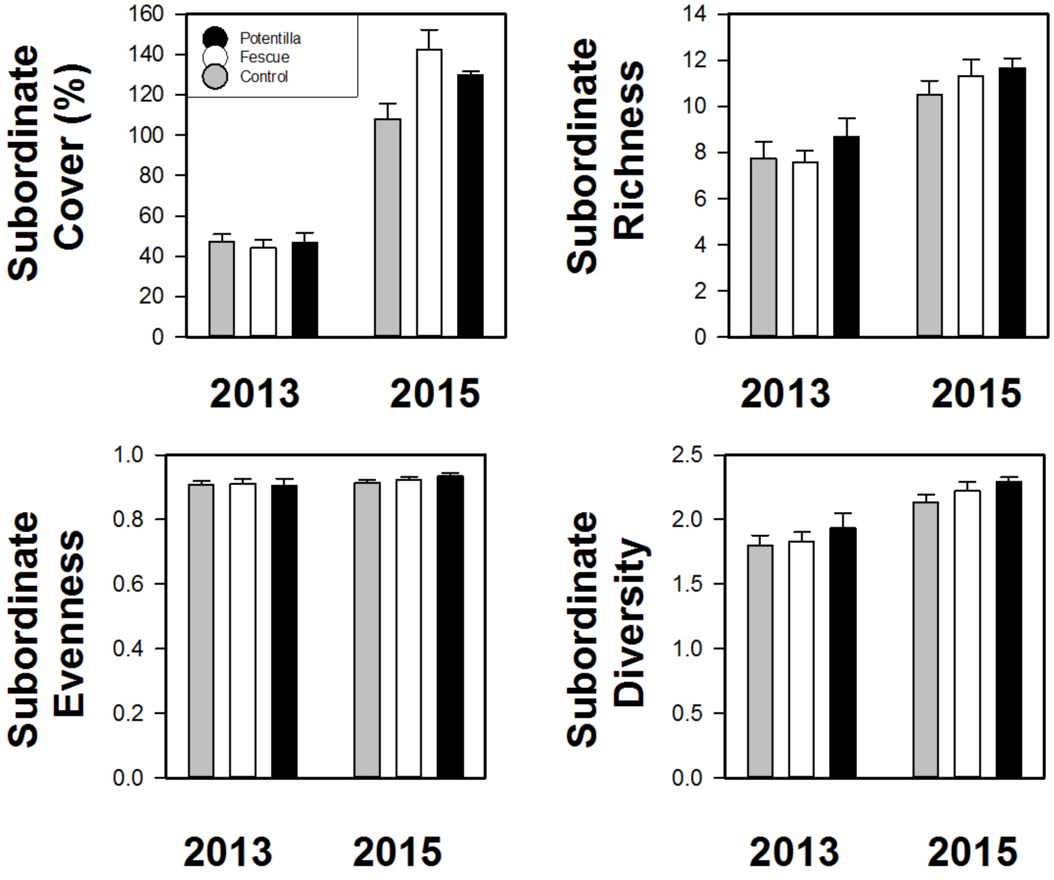

Supplementary Figure 2. Principal Coordinate Ordination (PCO) illustrating in a two-dimensional scale (PCO Axis 1 and PCO Axis 2) subordinate species composition across a plant removal treatments (*Potentilla* removal, *Festuca* removal, no removal control) for growing seasons 2013 and 2015.

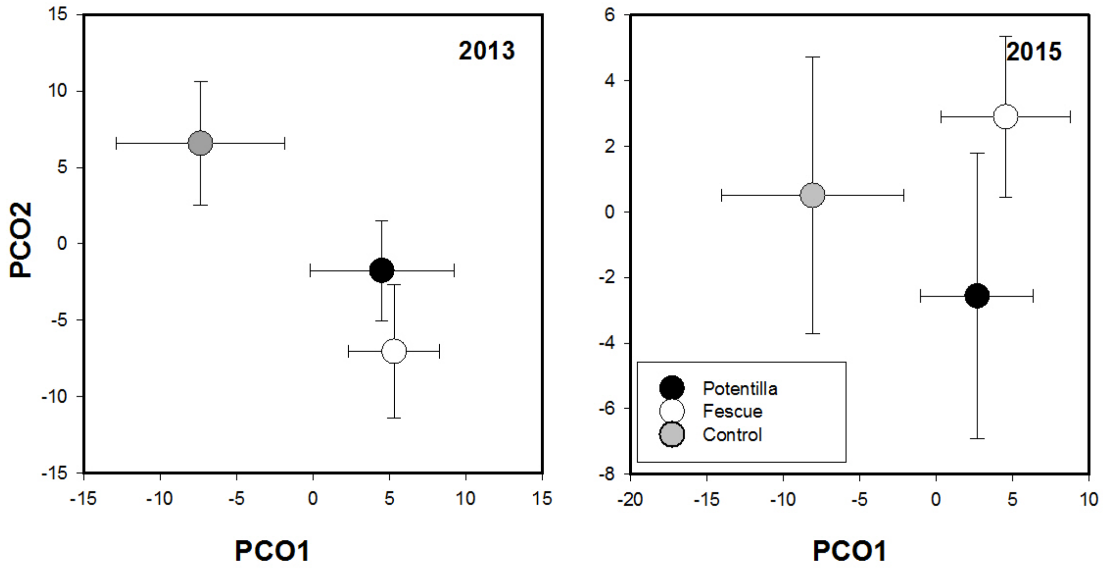

Appendix Table 1. Plant species list and dominance category for montane meadows used in observational and field experimental study. Dominance category was determined based on Mariotte et al. 2014.

Dominance Category	Species Identity	Dominance Category	Species Identity
Subordinate	*Achillea millefolium*	Transient	*Ipomopsis aggregata*
Transient	*Achnatherum pinetorum*	Subordinate	*Lathyrus leucanthus*
Transient	*Agoseris aurantiaca*	Transient	*Linum lewisii*
Transient	*Androsace chamaejasme*	Transient	*Oxalis stricta*
Transient	*Artemisia ludiciviana*	Transient	*Oligosporus dracunculus*
Transient	*Bromalis spectabilis*	Transient	*Rananculus inomaeus*
Subordinate	*Bromopsis inermis*	Transient	*Taraxacum officinale*
Transient	*Campanula douglasii*	Subordinate	*Thalictrum fendleri*
Transient	*Chenopodium douglasii*	Transient	*Tragopogon dubious*
Transient	*Collomia linearis*	Subordinate	*Vicia americana*
Transient	*Epilobium hornmannii*	Transient	*Viola nuttallii*
Dominant	*Erigeron speciosus*	Transient	*Viola scopulorum*
Dominant	*Festuca thurberi*	Transient	*Zigadin elegans*
Transient	*Fragaria Virginiana*	Transient	*Chenopodium sp.*
Subordinate	*Galium borealis*	Transient	*Eridgeron speciosa*
Transient	*Hackelia Florabanda*	Dominant	*Festuca thurberi*
Transient	*Helenium hoopesii*	Transient	*Ligusticum porteri*
Transient	*Heliomeris multiflora*	Transient	*Erigeron elatior*
Transient	*Helientella hopsii*	Transient	*Poa pratensis*
Transient	*Helianthella quinquenervis*	Dominant	*Potentilla gracilis*
